# Effects and cost-effectiveness of pharmacogenetic screening for CYP2D6 among older adults starting therapy with nortriptyline or venlafaxine: study protocol for a pragmatic randomized controlled trial (CYSCEtrial)

**DOI:** 10.1186/s13063-015-0561-0

**Published:** 2015-01-31

**Authors:** Elizabeth JJ Berm, Eelko Hak, Maarten Postma, Marjolein Boshuisen, Laura Breuning, Jacobus RBJ Brouwers, Ton Dhondt, Paul AF Jansen, Rob M Kok, Jan G Maring, Rob van Marum, Hans Mulder, Richard C Oude Voshaar, Arne J Risselada, Harry Venema, Liesbeth Vleugel, Bob Wilffert

**Affiliations:** Groningen Institute of Pharmacy, University of Groningen, Unit of Pharmacotherapy & Pharmaceutical Care, Antonius Deusinglaan 1, 9713AV Groningen, The Netherlands; Groningen Institute of Pharmacy, University of Groningen, Unit of Pharmacoepidemiology & Pharmacoeconomics, Groningen, The Netherlands; Department of Epidemiology, University Medical Center Groningen, Groningen, The Netherlands; Lentis, Dignis, Groningen, The Netherlands; Departement of Old Age and Clinical Psychiatry, Reinier van Arkel Group, ‘s-Hertogenbosch, The Netherlands; Department of Geriatric Medicine and Expertise Centere Pharmacotherapy in Old Persons, UMC Utrecht, Utrecht, The Netherlands; Department of Old-age Psychiatry, GGZ-Noord Holland Noord, Heerhugowaard, The Netherlands; Department of Old Age, Parnassia Psychiatric Institute, The Hague, The Netherlands; Laboratory for Drug Analysis & Toxicology, Diaconessen Hospital Meppel & Bethesda Hospital Hoogeveen, Meppel, The Netherlands; Department of Geriatric Medicine, Jeroen Bosch Hospital, ‘s Hertogenbosch, The Netherlands; Department of General Practice & Elderly Care Medicine, VU University, Amsterdam, The Netherlands; Clinical Pharmacy, Wilhelmina Hospital Assen, Assen, The Netherlands; University of Groningen, University Medical Center Groningen, University Center for Psychiatry, Groningen, The Netherlands; Department of Old Age Psychiatry, GGZ Friesland, Leeuwarden, The Netherlands; Department of Old Age Psychiatry, GGZ inGeest, Haarlem, The Netherlands; Department of Clinical Pharmacy and Pharmacology, University Medical Center Groningen, Groningen, The Netherlands

**Keywords:** CYP2D6, Pharmacogenetics, Depression, Nortriptyline, Venlafaxine

## Abstract

**Background:**

Nortriptyline and venlafaxine are commonly used antidepressants for treatment of depression in older patients. Both drugs are metabolized by the polymorphic cytochrome P450-2D6 (CYP2D6) enzyme and guidelines for dose adaptations based on the *CYP2D6* genotype have been developed. The CYP2D6 Screening Among Elderly (CYSCE) trial is designed to address the potential health and economic value of genotyping for *CYP2D6* in optimizing dose-finding of nortriptyline and venlafaxine.

**Methods/Design:**

In a pragmatic randomized controlled trial, patients diagnosed with a major depressive disorder according to the DSM-IV and aged 60 years or older will be recruited from psychiatric centers across the Netherlands. After *CYP2D6* genotyping determined in peripheral blood obtained by finger-prick, patients will be grouped into poor, intermediate, extensive, or ultrarapid metabolizers. Patients with deviant genotype (that is poor, intermediate or ultrarapid genotype) will be randomly allocated to an intervention group in which the genotype and dosing advice is communicated to the treating physician, or to a control group in which patients receive care as usual. Additionally, an external reference group of patients with the extensive metabolizer genotype is included. Primary outcome in all groups is time needed to obtain an adequate blood level of the antidepressant drug. Secondary outcomes include adverse drug reactions measured by a shortened Antidepressant Side-Effects Checklist (ASEC), and cost-effectiveness of the screening.

**Discussion:**

Results of this trial will guide policy-making with regard to pharmacogenetic screening prior to treatment with nortriptyline or venlafaxine among older patients with depression.

**Trial registration:**

ClinicalTrials.gov: NCT01778907; registration date: 22 January 2013.

**Electronic supplementary material:**

The online version of this article (doi:10.1186/s13063-015-0561-0) contains supplementary material, which is available to authorized users.

## Background

Depressive disorder is a chronic disease with a considerable impact on mental and physical health as well as quality of life [1]. The 1-month prevalence of major depressive disorder (MDD) in the Dutch population (aged 55 to 85 years) is estimated around 2.0% [2]. From a meta-analysis (studies included from 1999 to 2009) a higher point prevalence estimation of 7.2 % has been reported for late life depression (75+ years) [3]. Among all mental diseases, MDD is ranked as a very disabling disease [4] and depressive disorders are projected to be the largest cause for disability by 2030 in high-income countries [5].

MDD can be treated with antidepressants, which are classified into different groups: selective serotonin reuptake inhibitors (SSRIs), tricyclic antidepressant (TCAs) and others (for example,. selective serotonin-noradrenaline reuptake inhibitors (SNRIs), mirtazapine, monoamine oxidase inhibitors). Although overall efficacy estimates are virtually similar between these drug groups, adverse drug profiles differ [6]. Worldwide, clinical guidelines usually advise first-step treatment with an SSRI because of a more favorable adverse effect profile compared to other antidepressants (for example, fewer cardiac side-effects and often lower drug anticholinergic side-effects) [7,8]. If the chosen SSRI is not effective, switching to a TCA, preferably nortriptyline, or an SNRI, mostly venlafaxine, is an option [7].

Venlafaxine and nortriptyline are both metabolized by the polymorphic cytochrome P450 (CYP2D6) iso-enzyme [8]. Its polymorphic differences are generally classified into four different phenotypes: poor metabolizers (PM), intermediate metabolizers (IM), extensive metabolizers (EM), and ultrarapid metabolizers (UM). With respect to EM, PM and IM have a decreased enzymatic activity whereas UM have a higher activity, which is described in more depth elsewhere [9]. As a result of the variation in metabolizing capacity, as well as differences in gender, age, co-medication, and comorbidity among patients, different pharmacokinetics are observed [9,10]. These differences can be monitored by therapeutic drug monitoring (TDM), which helps to find the right dosage for an individual patient. TDM of nortriptyline is ‘strongly recommended’ and for venlafaxine it is recommended especially during start of therapy [11]. Prior and in addition to TDM, information about the metabolic CYP2D6 activity could further improve this dose-finding. Since it is possible to detect polymorphic differences of *CYP2D6* based on genetic material, it is not dependent on the actual antidepressant intake (patient compliance) or collection time of the sample, like TDM samples are.

For nortriptyline and venlafaxine genotyped-based dose adjustment guidelines have been formulated [10,12,13]. In the Netherlands, the Royal Dutch Pharmacist Association (KNMP) has made such guidelines available for current clinical practice [14,15].

Despite the large number of studies that assessed the relationship between pharmacogenetics and pharmacokinetics of antidepressants, implementation of pharmacogenetic knowledge into clinical practice is still scarce. The hypothesized clinical improvements facilitated by genotype-based dose adjustments in addition to current TDM, like improved efficacy and prevention of adverse drug reactions, are still a matter of controversy and discussion [16,17]. There is a need to test this hypothesis in daily clinical practice. Therefore, we designed a pragmatic multicenter randomized controlled trial to determine the effects of a pharmacogenetic screening for *CYP2D6* on the time needed to obtain adequate blood levels of nortriptyline or venlafaxine. Since older persons are more vulnerable for adverse drug reactions, beneficial effects of genotype adjusted dosing are expected to be more apparent in this population. Therefore, the trial is conducted among older depressed patients starting with nortriptyline or venlafaxine. The cost-effectiveness of this screening will be assessed to support decision-making on the potential implementation of screening for *CYP2D6* genotype in daily clinical practice. The trial is designed to allow reporting according to the CONSORT guidelines [18].

## Method/Design

### Study design

**Figure 1:**
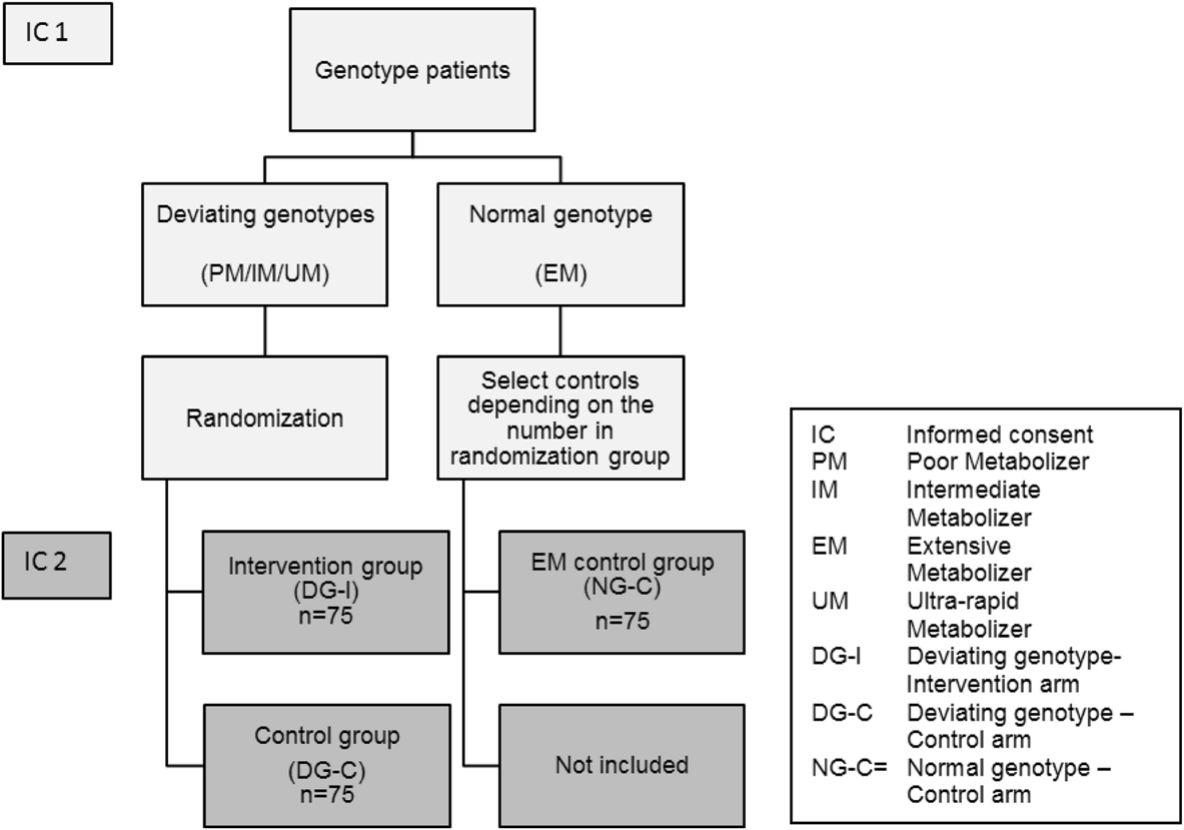
**Flowchart for inclusion of patients.**

The study is a multicenter randomized controlled trial across multiple old age psychiatry and geriatric mental health care institutions in the Netherlands. Patients will be recruited by their treating physician or a specialized trial nurse. The study consists of two parts and for both parts separate written informed consent will be obtained. The first part is a basic genotype screening study in which each eligible patient (see description in Participants below) starting with nortriptyline or venlafaxine will be asked permission for genotyping. Patients with a PM, IM and UM genotype will be selected for participation in the second (trial) part of the study. After giving the second informed consent, the trial entails a follow-up period of 6 weeks or longer in order to monitor the blood levels and adverse reactions of either nortriptyline or venlafaxine. A random selection of patients with an EM genotype will be allocated to an additional reference group (Figure 1). Recruitment is expected to be completed within approximately 3 years. If selected for the trial part of the study, eligible patients are invited for a baseline visit. At baseline, 2 weeks, and 4 weeks after baseline blood samples are collected to estimate the blood level of the drug by a ‘Dried Blood Spot’ (DBS) method. Additionally, questionnaires concerning adverse drug reactions, quality of life, productivity and health care use, and severity of depression are completed. Patients who do not complete dose-finding after 6 weeks will be followed for additional 2-week measurements until dose-finding is completed. These additional 2-week samples will be collected only for those patients who had a dose change 3 weeks or less prior to the moment the sample was collected, since it can take up to 2 to 3 weeks for PMs of nortriptyline to reach steady state concentration [19]. When the follow-up period is finished for an individual patient, the genotype information will be supplied to the treating physician. Ethical approval was obtained by an Independent Ethics Committee (RTPO-Leeuwarden-NL; file number: NL40925.099.12) and the study will be conducted in accordance with the Declaration of Helsinki.

### Participants

Patients 60 years or older and diagnosed with a major depression according to the *Diagnostic and Statistical Manual of Mental Disorders, fourth edition text revision* (DSM-IV TR) [20], criteria (code: 296.2x or 296.3x) diagnosed by the treating physician, are eligible for inclusion. Patients should be starting with either nortriptyline or venlafaxine and competent to understand the two separate informed consent procedures. Patients with known liver cell damage, which in clinical practice often serves as a proxy for a poor hepatic function (aspartate aminotransferase and alanine aminotransferase (ASAT/ALAT) or gamma-glutamyl transferase (γ-GT) ≥ twice the maximal reference value), impaired renal function (eGFR < 30 mL/minute) in combination with venlafaxine use, or currently using drugs influencing blood levels of nortriptyline or venlafaxine are excluded for trial participation. Patients using terbinafine, ketoconazole, voriconazole, kinidine, propafenon, cimetidine, fluoxetine, paroxetine, bupropion, duloxetine, sertraline, abirateron, cinacalcet, rifampicine, and ritanovir are excluded for trial participation. For these drugs, interactions can be expected based on pharmacy interaction monitoring software (G-standaard, Z-index BV, The Hague, The Netherlands), summary of product characteristics, and Flockhart’s interaction table [21].

#### *CYP2D6* genotyping

In the first part of the study, a DBS sample for genotyping will be taken at the time of prescription of nortriptyline or venlafaxine by means of a finger-prick [22]. The sample will be sent to the Pharmacogenetic lab of the Wilhelmina Hospital Assen (Assen, the Netherlands), for analysis. Single nuclear polymorphism (SNPs) will be assessed for the *3, *4, *5, *6, *10, *17, *41 alleles, as well as duplications of the *CYP2D6* gene. Presence of an allele that completely lacks enzyme activity (*3, *4, *5, *6 ) paired with another allele completely lacking enzyme activity results in a PM phenotype prediction and in an IM phenotype prediction if paired with an allele with decreased enzyme activity (*10, *17, *41). Presence of only one allele lacking enzyme activity, or two alleles with decreased enzyme activity also results in an IM phenotype prediction. Duplications of the *CYP2D6* gene results in an UM phenotype prediction unless it is combined with any allele with reduced or lacking enzyme activity. Duplications combined with any allele with reduced or lacking enzyme activity results in exclusion of the patient from follow-up because no adequate dosing advice can be given for these genotypes. After inclusion, genotype information will be available for randomization within 6 to 9 days.

### Baseline

Baseline assessment will include a severity of depression score measured by the Montgomery-Asberg Depression Rating Scale and measurement of symptoms which are followed-up as adverse events during the further trial [23]. In addition, comorbidities and drug use other than nortriptyline and venlafaxine will be collected by the Short Form Health and Labor Questionnaire [24].

### Randomization and allocation

After the genotype is determined, patients with a PM, IM, or UM genotype are selected and randomly allocated by computer to the ‘deviating genotype-intervention arm’ (DG-I) or ‘deviating genotype-control arm’ (DG-C) for participation in the main study (Figure 1). An additional sample of patients with the EM genotype is allocated to a third ‘normal genotype- control’ arm (NG-C). The PM, IM and UM genotypes for *CYP2D6* are expected to be found in approximately 30% of the population [9]. Therefore, it is expected that patients with the EM genotype will be found more frequently than patients with a deviating genotype. To prevent any potential time-dependent bias, the selection of patients with an EM genotype allocated to the NG-C arm is dependent on the number of patients in the other trial arms.

#### Deviating genotype-intervention arm (DG-I)

The DG-I arm includes patients with a PM, IM, or UM genotype. The specific genotype accompanied by dosing advice is directly communicated to the treating physician. This should preferably take place 14 days after inclusion. The dosing advice is based on the genotype and drug only, meaning some standard advice depending on these two variables is given by an automatically generated message (see Additional file 1).

#### Deviating genotype-control arm (DG-C) and normal genotype-control arm (NG-C)

The DG-C arm includes patients with a deviating genotype and the NG-C arm will include patients with a normal genotype. In contrast to patients in the intervention arm, the treating physician will not be informed about the genotype of patients in one of the control arms. Although methodologically there is a different approach for deviating and normal genotype patients, for the physician, patients will appears as one control group. This ensures that patients in the control group can have any genotype as in the normal population.

### Outcomes

#### Primary outcome

Primary outcome will be the time needed to reach adequate blood levels of nortriptyline or venlafaxine, which will be determined by a DBS sample [25,26]. The use of DBS for therapeutic drug monitoring is described in more depth elsewhere [27]. Blood samples will be sent to the Laboratory for Drug Analysis & Toxicology of the Diaconessen Hospital (Meppel, the Netherlands). Advised therapeutic ranges follow the current Dutch Clinical Pharmacy guidelines (NVZA), indicating for nortriptyline levels between 50 to 150 μg/L and for venlafaxine + O-desmethylvenlafaxine between 100 to 400 μg/L. We defined adequate drug levels as [1] being within the therapeutic range and [2] no dose changes during the previous 3 weeks.

#### Secondary outcome

Secondary outcomes will be adverse drug reactions measured by a shorter and modified version of the Antidepressant Side-Effect Checklist (ASEC) [28], quality of life measured by the EuroQol 5D (EQ5D) [29], and productivity and cost of health care use measured by the Trimbos/iMTA questionnaire for Cost associated with Psychiatric Illness (TiC-P) [24]. Severity of depression will be measured to control for possible differences between groups related to the severity of depression instead of the genotype-based intervention, by the Quick Inventory of Depressive Symptomatology Self-Reported Questionnaire (QIDS-SR) [30]. Secondary outcomes will all be measured by a telephone interview from baseline till endpoint, every 2 weeks, except for the side-effects. Side-effects will be assessed by the treating physician and will additionally be collected before start of the treatment. In this way we will be able to correct for symptoms of depression or other underlying physical diseases, which are often perceived by the patient as side-effects of the antidepressant [28].

### Sample size calculation

Based on clinical experience in the research setting it is expected that on average it takes 4 weeks for patients with a deviating genotype to reach adequate serum drug levels. It is hypothesized that time to reach adequate levels will be reduced by 50% (2 weeks) if we provide the screening information to the physician within 6 to 9 days. To obtain 90% power to detect a 50% reduction from 4 to 2 weeks (SD 3 weeks) with a type-1 error of 2.5% to account for multiple testing (within trial and externaltrial), a minimum of 48 patients per arm is needed. To account for loss to follow-up we intend to include at least 75 patients per arm.

### Statistical analysis and report

Reporting and analysis of the data from this trial will be in accordance with the CONSORT 2010 guidelines [18].

#### Baseline

Baseline characteristics will be presented using descriptive statistics including means or medians for continuous variables and percentages for categorical variables. Analytical statistics to estimate difference between the group of patients who were lost to follow-up versus patients who completed the trial as well as between the different study arms will be determined by *t*-tests or non-parametric alternatives for continuous variables and Chi-square tests for categorical variables.

#### Primary analysis

The primary analysis will assess the mean time needed to obtain adequate drug levels. The first time drug blood levels are within the therapeutic range and dose is not increased or decreased in the following 3 weeks is the moment that is considered as the time needed to obtain adequate drug levels. Mean differences in time to reach adequate drug levels between trial groups will be estimated using the *t*-test or a non-parametric alternative if not normally distributed. Analysis of variance (ANOVA) or Kruskal-Wallis test will be applied to test for potential differences between the trial groups and the external reference group.

#### Secondary analyses

Secondary analysis will focus on statistical differences in median number and severity scores of side-effects of the drug. Since the side-effect profile is different for nortriptyline and venlafaxine, these analyses will be drug-specific.

#### Cost-effectiveness analyses

Effects and costs will be determined from a societal perspective including both indirect and direct costs. Data on health care associated resource use will be collected and effects on productivity and quality of life (EQ5D) will be investigated. To determine the incremental cost-effectiveness ratio for intervention versus control strategy from a societal perspective, guideline unit costs will be linked to these resource use data. Incremental cost-effectiveness will be calculated using state-of-the-art methods including uncertainty analysis (bootstrapping, Fieller’s estimates), scenario analysis, (probabilistic) sensitivity analysis and presentation in cost-effectiveness acceptability curves.

## Discussion

To the best of our knowledge, this is the first pragmatic randomized controlled trial designed to test whether *CYP2D6* polymorphism genotyping among older depressed patients starting with nortriptyline or venlafaxine in clinical practice adds value to regular care. Results of this trial will generate evidence as to whether routinely-based genotype testing can reduce the time needed for dose-finding at the start of treatment and as a result reduce adverse drug reactions.

A possible limitation of this study is that the genotype information is given to the physician after treatment with the antidepressant has already been started for several days. For the purpose of this trial, it would be ideal to give the genotype information before treatment has started. However, in clinical practice due to the urgency of the disease, time between initiating a therapy with nortriptyline or venlafaxine and actual start is limited. Waiting for the genotype information before starting pharmacotherapy could delay pharmacotherapy and is, therefore, unwanted. Nevertheless, an effect of genotype is still expected, because at start of treatment a low dose is generally prescribed. When the treating physician has to evaluate therapy and to decide if the dosage should be increased, the genotype information will be available [31].

Another limitation of this study is the study domain represented by a heterogeneous group of severely depressed patients, since the disease shows much variation in symptomatology, severity and co-existing psychiatric diseases like anxiety disorders and physical diseases, especially in secondary care [32]. However, a strict selection of patients who suffer from depression without the presence of certain comorbid physical or psychiatric disorders would exclude a lot of patients from this study and limit applicability of the results to daily practice. Therefore, inclusion is not limited by severity of depression, co-existing psychiatric diseases or other comorbidities. This is a common characteristic of pragmatic trials and gives results which are better generalizable to actual clinical practice [33].

Furthermore, the drug concentration-effect relationship for venlafaxine is still a subject of debate [34]. This is illustrated by different advised therapeutic windows. Some refer to a therapeutic window between 250 to 750 μg/L, whereas recent guidelines advice a therapeutic window between 100 to 400 μg/L, which is also incorporated in current Dutch guidelines [11,35]. Nevertheless, current guidelines advise a smaller therapeutic window, indicating the need for a more accurate drug dosing. To facilitate this, genotyping may play an important role.

Strengths of this study are the random allocation of subjects to receive genetic information that avoids incomparability of groups, strict protocolled design with regular measurements, and a large number of patients to detect statistically significant differences. We also included an external control arm (NG-C) including patients with the EM genotype. This ensures physicians do not know the genotype of control patients or that it deviates from EM.

The overall impact of *CYP2D6* screening may be underestimated in this trial, since the genotype is a lifelong characteristic and is, therefore, affecting the metabolism of many other drugs than the drugs studied in this trial. We only look at a short drug-specific time effect in this study; however, it is not unlikely that patients might benefit from their genotype information during further pharmacotherapy.

## Trial status

The recruitment of patients started in spring 2013 and is expected to continue until spring 2016.

## Additional file

Additional file 1:
**Advice based on the Dutch KNMP’s pharmacogenetic guidelines, specified for this specific study purpose with the help of experts in the field.** Description: this file contains the advice that was communicated to the treating physicians during the study period.

## Abbreviations

ALT: alanine aminotransferase
ANOVA: analysis of variance
ASEC: Antidepressants Side-Effect Checklist
AST: aspartate aminotransferase
DBS: Dried Blood Spot
DG-C: deviating genotype control
DG-I: deviating genotype intervention
DSM: *Diagnostic and Statistical Manual of Mental Disorders*
EQ5D: EuroQol 5D quality of life quesionnaire
EM: extensive metabolizer
γGT: gamma-glutamyl transferase
IM: intermediate metabolizer
KNMP: Royal Dutch Pharmacist Association
NG-EC: normal genotype-external control
MDD: major depressive disorder
NVZA: Dutch Clinical Pharmacy guidelines
PM: poor metabolizer
QIDS-SR: Quick Inventory of Depressive Symptomatology Self-Reported Questionnaire
SNRI: selective serotonin-noradrenaline reuptake inhibitor
SSRI: selective serotonin reuptake inhibitor
SNP: single nuclear polymorphism
TCA: tricyclic antidepressant
TiC-P: Cost associated with Psychiatric Illness
TDM: therapeutic drug monitoring
UM: ultrarapid metabolizer
