# Supplementary material for: Effects and cost-effectiveness of pharmacogenetic screening for CYP2D6 among older adults starting therapy with nortriptyline or venlafaxine: study protocol for a pragmatic randomized controlled trial (CYSCEtrial)
Source: Trials. 2015 Jan 31;16:37. doi: 10.1186/s13063-015-0561-0 (PMC4328880; doi:10.1186/s13063-015-0561-0)
Supplement: Additional file 1: — Advice based on the Dutch KNMP’s pharmacogenetic guidelines, specified for this specific study purpose with the help of experts in the field. Description: this file contains the advice that was communicated to the treating physicians during the study period. [file 13063_2015_561_MOESM1_ESM.docx]

**Appendix 1. Advices based on the Dutch KNMP-pharmacogenetic guidelines, specified for this specific study purpose with the help of experts in the field**

| *Genotype* | *Nortriptyline* | *Venlafaxine* |
| --- | --- | --- |
| PM/ intervention group | During genotyping a deviating genotype was found. This leads to classification of the patient as a Poor Metabolizer (PM). A PM has a lower capacity of the CYP2D6 enzyme which can cause an increase in nortriptyline plasma concentrations.  Advice: give a 50% lower dosage with respect to normal dosing.  Additional information: For a PM dosing between 50 and 75 mg /day should give blood levels within the therapeutic window. However, differences between individuals can be significant. Dosing above 75 mg/day gives a substantial chance of blood levels exceeding the therapeutic window. The half-live of the drug is extended. Keep in mind that it can take longer before a steady state period is reached (12-21 days, normal 7-8 days). | During genotyping a deviating genotype was found. This leads to classification of the patient as a Poor Metabolizer (PM). A PM has a lower capacity of the CYP2D6 enzyme which can cause an increase in venlafaxine (V) plasma concentrations and a decrease of its active metabolite *o*-desmethylvenlafaxine (ODV). There is evidence suggesting a reduced efficacy among depressed patients with this genotype.  Advice: In case of response combined with adverse drug reactions: lower dosing to 75% of normal dosing. This should give a sum of V+ODV equal to that of an extensive metabolizer (EM). However, differences between individuals are significant and the ratio V/ODV remains different from an EM. If dosing is further decreased it is unknown to which extend efficacy is maintained. |
| IM/ intervention group | During genotyping a deviating genotype was found. This leads to classification of the patient as an Intermediate Metabolizer (IM). An IM has a lower capacity of the CYP2D6 enzyme which can cause an increase in nortriptyline plasma concentrations.  Advice: give a 75% lower dosage with respect to normal dosing.  Additional information: For a IM dosing 75 mg /day should give blood levels within the therapeutic window. However, differences between individuals can be significant. Dosing above 75 mg/day should preferably be guided by TDM. The half-live of the drug is probably extended. Keep in mind that it can take longer before a steady state period is reached (9-21 days, normal 7-8 days). | During genotyping a deviating genotype was found. This leads to classification of the patient as an Intermediate Metabolizer (IM). An IM has a lower capacity of the CYP2D6 enzyme which can cause an increase in venlafaxine (V) plasma concentrations and a decrease of its active metabolite *O*-desmethylvenlafaxine (ODV). Kinetic profiles show an increase in V+ODV concentration of 1-22%. There is insufficient evidence to give a dose adaptation based on literature. The advice is to control the sum of V + ODV by TDM and adapt dosing if indicated.  Although there is no dosing advice available, the patient remains in the trial. This is to collect more data concerning elderly and the IM genotype. |
| UM/ intervention group | During genotyping a deviating genotype was found. This leads to classification of the patient as an Ultrarapid Metabolizer (UM). A UM has an increased capacity of the CYP2D6 enzyme which can cause a decreased nortriptyline plasma concentration and an increased plasma concentration of metabolites (E-10-OH-nortriptyline).  Advice: Increase dosing to 150%. The metabolite of nortriptyline (OH-nortriptyline) might be cardiotoxic. Be alert to changes in the EKG.  Additional information:  For an UM dosing of 100 mg can give therapeutic concentrations. To reach adequate therapeutic levels dosing up to 300 mg/day can be necessary. However, differences between individuals are significant. The half-life of the drug is decreased. Keep in mind steady state is reached earlier (4-7 days, normal 7-8 days). | During genotyping a deviating genotype was found. This leads to classification of the patient as an Ultrarapid Metabolizer (UM). A UM has an increased capacity of the CYP2D6 enzyme which can cause a decreased venlafaxine plasma concentrations and an increased plasma concentration of the active metabolite *O*-desmethylvenlafaxine.  Advice: Be alert to low sum plasma concentrations of V and ODV. If indicated by TDM increase dosing up to 150% of normal dosing. |
| EM/PM/IM/UM/ control group | The patient is not included in the intervention group. Therefore we ask you to continue care as usual. Knowing a patient is in the control group does not give you any information concerning the genotype of the patient, since patients with either a decreased, increased and normal metabolism are included in this group. | |
